# From Pig to Pacifier: Chitterling-Associated Yersiniosis Outbreak among Black Infants

**DOI:** 10.3201/eid0908.030103

**Published:** 2003-08

**Authors:** Timothy F. Jones, Steven C. Buckingham, Cheryl A. Bopp, Efrain Ribot, William Schaffner

**Affiliations:** * Tennessee Department of Health, Nashville, Tennessee, USA; † University of Tennessee Health Science Center, Memphis, Tennessee, USA; ‡ Le Bonheur Children's Medical Center, Memphis, Tennessee, USA; § Centers for Disease Control and Prevention, Atlanta, Georgia, USA; ¶ Vanderbilt University School of Medicine, Nashville, Tennessee, USA

**Keywords:** yersinia, outbreak, chitterlings, infant, dispatch

## Abstract

In this case-control study of *Yersinia enterocolitica* infections among black infants, chitterling preparation was significantly associated with illness (p<0.001). Of 13 samples of chitterlings tested, 2 were positive for *Yersinia intermedia* and 5 for *Salmonella*. Decontamination of chitterlings before sale with methods such as irradiation should be strongly considered.

*Yersinia enterocolitica* is an uncommon cause of illness outbreaks in the United States. Of 7,390 foodborne disease outbreaks reported to the Centers for Disease Control and Prevention (CDC) from 1990 through 1999, 5 (<0.1%) were reported to be caused by *Yersinia* (*1*). It is also a relatively uncommon cause of sporadic disease, accounting for <0.3% of all foodborne illness in the United States (*2*). *Y. enterocolitica* has been isolated from a variety of animal reservoirs, and outbreaks have been attributed to contaminated water, milk, bean sprouts, and pork intestines (*3*). We investigated an outbreak of gastrointestinal illness in black infants in Tennessee.

## The Study

Case-patients were defined as residents of Tennessee <1 year of age with culture-confirmed *Y. enterocolitica* infection occurring from November 15, 2001 to February 15, 2002. A case-control study was performed to define risk factors for infection. Controls were randomly selected from a list of black patients <1 year of age who were evaluated in the emergency department of the large urban children’s hospital where the outbreak was initially identified, with any diagnoses other than *Yersinia* gastroenteritis during the outbreak period. A structured questionnaire was administered to patients and controls by telephone. Both patients and controls were asked about exposures from November 1, 2001 to January 31, 2002.

Isolates of *Y. enterocolitica* from cases were confirmed, biotyped, and serotyped at CDC (*4*). Isolates were also tested for several biochemical markers of pathogenicity (*5*). All available isolates were subtyped by pulsed-field gel electrophoresis (PFGE) after restriction of the genomic DNA with *Bln*1 or *Not*1 (*6*). Samples of chitterlings purchased 2 months after the outbreak from grocery stores in two large urban areas of the state, including the city where most case-patients lived, were cultured for *Yersinia* and *Salmonella* (*4*).

**Figure 1:**
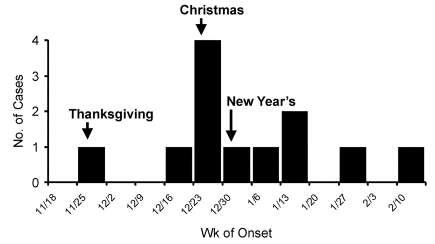
Epidemiologic curve of *Yersinia enterocolitica* outbreak in Tennessee.

Twelve cases of *Yersinia* infection in infants <1 year of age were identified in Tennessee with onset from November 15, 2001 to February 15, 2002 (Figure 1). All cases were identified by stool culture. Six cases occurred in December, and 10 were medically evaluated in the same city. All case-patients were black. In comparison, 49% of the population of the urban county in which the outbreak was identified is black.

Ten patients or caregivers and 51 controls were interviewed in the case-control study. Two infants with *Y. enterocolitica* infection could not be located. Of the 10 cases interviewed, 8 had received medical care in the same city, and 7 of those were seen at the same pediatric medical center. Median age of cases was 165 days (range 46–275 days), and median age of controls was 181 days. Four (40%) patients were hospitalized for their illness for a mean of 4 days. All patients had diarrhea, 7 (70%) reported bloody stools, 7 (70%) had vomiting, and 8 (80%) reported fever. Case-patients did not share apparent common exposures, such as childcare, social gatherings, grocery stores, or foods.

Chitterlings had been prepared in the homes of all case-patients from November 1, 2001, to January 31, 2002, compared with 35% of controls (p<0.001). The specific brand of chitterlings purchased could not be identified by 50% of infants’ caretakers. At least four different brands of chitterlings were purchased from at least five different grocery store chains by the families. The median time from preparation of chitterlings to onset of symptoms was 4 days (range 0–43). From 10 to 80 pounds of chitterlings were prepared at a time; chitterlings were thawed and cleaned in the kitchen sink over several hours. Parents of seven case-patients acknowledged exposures that could have led to infection of the infant. These exposures included the infant roaming freely in a walker in the room where chitterlings were being cleaned, chitterling juices splashing on clean dishes or a baby’s bottle, washing bottles in a sink that was not thoroughly cleaned, and feeding or handing a pacifier to an infant during cleaning or preparation of chitterlings.

**Figure 2:**
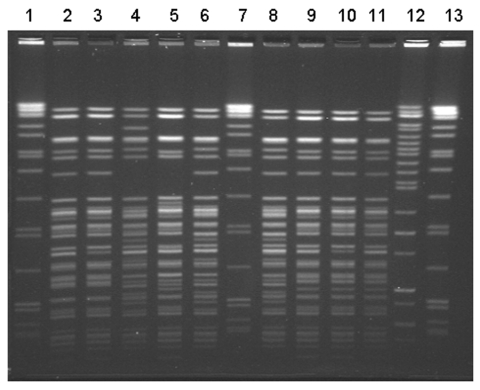
Pulsed-field gel electrophoresis (PFGE) patterns of *Yersinia enterocolitica* isolates from patients in this outbreak associated with chitterlings. Ten isolates from nine patients were available for typing. Seven distinct *Bln*I PFGE patterns were noted. Three infants had pattern #1 (lanes 9–11). Two infants shared pattern #2 (lanes 2 and 3). One patient had two distinct isolates (lanes 6 and 12). The molecular size standard is located in lanes 1, 7, and 13.

Nine *Yersinia* isolates from stool specimens of ill persons were available for further testing; all were identified as serotype O:3, biotype 4. Ten clinical isolates from nine case-patients were available for PFGE testing. Seven similar but distinct PFGE patterns differing by at least one band were noted (Figure 2). Three infants shared an indistinguishable pattern, and two other infants had another indistinguishable pattern. One case-patient had two isolates with distinct PFGE patterns. Of 13 samples of frozen chitterlings purchased in Tennessee in February, 2 were positive for *Y. intermedia*; 5 were positive for *Salmonella* (2 contained *S. Derby*, 1 *S.* Minnesota, and 2 *S.* Typhimurium var. Copenhagen).

## Conclusions

This outbreak of *Y. enterocolitica* infections affected black infants <1 year of age. Exposure to the preparation of chitterlings was a substantial risk factor for disease, though none of the infants directly ate chitterlings. No particular brand of chitterlings or specific preparation practice was implicated as the cause. The traditional holiday preparation of chitterlings involves a lengthy and messy process of cleaning and cooking raw pork intestines that may be fecally contaminated. Attempts to educate the public and change traditional methods of preparation (*7*,*8*) have been unsuccessful in preventing chitterling-associated outbreaks, and vulnerable “innocent bystanders” continue to be affected by the disease. Measures to eliminate contamination of chitterlings before sale should be developed to prevent disease among this high-risk population.

In many countries, *Y. enterocolitica* is a common cause of acute bacterial gastroenteritis, rivaling *Campylobacter* and *Shigella* in frequency (*9*). In the United States, *Y. enterocolitica* has been an uncommonly reported pathogen, although in recent years it has emerged as an occasional cause of sporadic illness and foodborne disease outbreaks. Outbreaks have been associated with water, contaminated milk, bean sprouts, tofu, and chitterlings (*8*–*10*). During a 7-year period at a hospital in Michigan, *Yersinia* accounted for 12.6% of bacterial intestinal pathogens isolated, with a rate comparable to *Campylobacter*. In that study, 99% of patients with *Yersinia* were black and 85% were infants; most illnesses occurred between November and January (*11*). At a large hospital in Georgia, *Y. enterocolitica* was isolated from 1% of rectal swabs submitted for culture. Among infants, *Y. enterocolitica* was second in frequency only to *Salmonella*, with a predominance in winter months (*12*).

Active laboratory-based surveillance for *Y. enterocolitica* infections in five sites in the United States demonstrated that the incidence in infants <1 year of age was more than 40-fold higher than the incidence in older age groups (SM Ray et. al, unpub. data). Furthermore, the incidence of *Yersinia* infection in blacks was more than seven times the incidence rate in non-blacks. A seasonal variation in incidence, with a marked peak in December, was noted only among blacks.

Since the 1980s, serogroup O:3 has replaced O:8 as the predominant serotype of *Y. enterocolitica* reported to CDC (*10*). Swine are the major reservoir of this serogroup (*3*,*13*), and the emergence of *Y. enterocolitica* infections in the United States has been attributed to the establishment of a widely distributed swine reservoir here (*8*).

This outbreak did not appear to be caused by a single brand or distributor of chitterlings. No apparent epidemiologic links existed between cases. The hospital at which the outbreak was recognized is the primary hospital for children in the area. In many black households, chitterlings are prepared annually by traditional methods. We were unable to identify any recent change in stool-testing procedures, products, or handling that would have explained an outbreak this year, in comparison with prior years, when similar methods were presumably used. The outbreak was recognized during active surveillance for foodborne pathogens at a hospital laboratory, as part of a program begun approximately 2 years ago. Earlier seasonal increases of *Y. enterocolitica* in this population may have gone unrecognized or unreported.

Chitterlings, prepared by boiling the large intestines of pigs after the removal of fat and fecal material, are a traditional winter-holiday food in many black families. In this outbreak, all affected persons were black infants. While none of the infants ate chitterlings, all were potentially exposed in homes where cleaning and preparation of chitterlings occurred. Because chitterlings are traditionally thoroughly boiled, the final cooked product is likely not to be bacterially contaminated. The preparation process, however, involves substantial handling of large amounts of potentially contaminated product, and the risk for exposure of infants to this process is high. This outbreak underscores the importance of rigid adherence to strict hygiene measures during handling of potentially contaminated foods. While we isolated a nonpathogenic *Yersinia* species from chitterling samples, this and other studies (*7*,*10*) demonstrate that chitterlings are not infrequently contaminated with enteric bacteria when purchased. Unfortunately, attempts to disseminate education regarding the safe preparation of chitterlings (*7*,*8*,*14*) have not prevented continued outbreaks among this high-risk population.

Irradiation of ground beef and other products can markedly reduce contamination with bacterial pathogens and improve food safety (*15*). Additional research is necessary to better understand barriers to acceptance of food irradiation among consumers and producers. Chitterlings are often purchased frozen in large containers and may be amenable to irradiation before sale. Irradiation should be studied as a potential means of preventing recurrent outbreaks among a vulnerable population of infants.
